# A Prospective Study of Immune Response After COVID-19 or Vaccination and Correlation Between Avidity Index and Neutralizing Capacity

**DOI:** 10.1155/av/2265813

**Published:** 2025-06-16

**Authors:** Emma Löfström, Anna Eringfält, Arne Kötz, Wanda Christ, Stefan Kunkel, Johan Tham, Jonas Klingström, Johan Undén

**Affiliations:** ^1^Department of Clinical Sciences, Lund University, Office DCSL, Lund 221 84, Sweden; ^2^Department of Clinical Microbiology, Hallands Hospital Halmstad, Halmstad 301 85, Sweden; ^3^Department of Medicine Huddinge, Center for Infectious Medicine, Karolinska Institutet, Stockholm 14186, Sweden; ^4^Department of Research and Development, Hallands Hospital Halmstad, Halmstad 301 85, Sweden; ^5^Department of Translational Medicine, Clinical Infection Medicine, Skåne University Hospital, Lund University, Malmö 205 02, Sweden; ^6^Department of Biomedical and Clinical Sciences, Linköping University, Linköping 581 83, Sweden; ^7^Department of Operation and Intensive Care, Hallands Hospital Halmstad, Halmstad 301 85, Sweden; ^8^Department of Clinical Sciences, Clinical Research Centre, Lund University, P.O. Box 50332, Malmö 20213, Sweden

**Keywords:** anti-spike IgG, avidity, COVID-19, immune response, neutralizing titer, SARS-CoV-2

## Abstract

**Background:** Serological response is an important aspect of COVID-19, especially for evaluation of vaccine effect and risk of severe infection. The gold standard to assess this is analysis of neutralization capacity, but such assays are not widely available, with antibody levels often used as an approximation of the neutralizing ability. Avidity index is a measure of antibody function and avidity maturation contributes to long-lasting immunity.

**Objectives:** To evaluate if the avidity index gives a better estimation of the neutralizing ability compared with anti-spike IgG levels and to compare the immune response between infection and vaccination against SARS-CoV-2.

**Study Design:** Serum samples from prospectively included patients with either PCR-confirmed COVID-19 or COVID-19-naïve after vaccination were analyzed for anti-spike IgG, avidity index, and neutralizing titer at 1, 3, and 6 months after infection or vaccination.

**Results:** A significant correlation between anti-spike IgG level and neutralization titer was found (Spearman*r*_*s*_ = 0.88, *p* < 0.001), but for the avidity index, the correlation was lower (Spearman *r*_*s*_ = 0.62, *p* < 0.001). Anti-spike IgG level, avidity index, and neutralization titer were significantly higher in the vaccine cohort. Natural infection failed to yield high-avidity antibodies. Over time, the antibody levels and neutralization titer declined in both the vaccine and infection cohorts.

**Conclusion: **Anti-spike IgG levels can be used as an estimation of the neutralizing titer, with the avidity index not contributing to a better estimation. There is a stronger initial immune response after vaccination, compared with natural infection. However, specific antibody levels decline over time, highlighting the importance of vaccine boosters.

## 1. Introduction

During the coronavirus disease (COVID-19) pandemic, the importance of immunity to this novel virus became evident. The development of a vaccine, as well as the development of natural immunity, against severe acute respiratory syndrome coronavirus 2 (SARS-CoV-2) has contributed to the end of the pandemic era [1]. However, COVID-19 can still cause severe illness, especially in immunocompromised patients where vaccines and previous infections have limited immunological effect [2]. Also, new virus variants are emerging, which highlights the importance of a long-lasting and protective immunity [3]. In order to help predict vaccine effect and the risk of severe illness in individual patients, there is a need for a simple and widely available method to assess immunity.

Analyzing the levels of antibodies with a commercial enzyme immune assay (EiA) is a common and relatively inexpensive method to estimate both vaccine effect and natural immunity in everyday clinical practice. However, this only reports the quantitative levels of antibodies and gives no information of the qualitative function. The gold standard to assess the actual function of antibodies is to assess the neutralizing capacity of the appropriate virus. However, these neutralizing assays are time consuming and require biosafety level 3 laboratories, as they use living viruses in cell cultures [4]. These assays are therefore unavailable in everyday clinical practice.

An often overlooked aspect of antibody function is avidity. The avidity is defined as the overall affinity, or the functional affinity, of the antibodies in sera and it can easily be analyzed by using a regular EiA and adding an extra step with urea as a chaotropic agent [5]. For some viral infections, there is a strong correlation between the avidity of the antibodies and the neutralization capacity, with the avidity index suggested as a surrogate marker for neutralization titer [6].

Since avidity maturation results from continuous evolution of B-cells, it contributes to long-lasting and protective immunity against several viruses [7–9]. Freitas et al. showed that high-avidity antibodies against respiratory syncytial virus (RSV) led to better protection and less risk of severe infection [10]. For mumps, Mercader et al. showed that incomplete avidity maturing after vaccination led to a higher risk of infection and Kontio et al. showed that not only decreasing levels of antibodies, but also decreasing avidity, can lead to measles and mumps infection in MMR-vaccinated individuals [11, 12]. For SARS-CoV-2, studies have showed failure of avidity maturation after natural infection but development of high avidity antibodies after vaccination [13–16]. This distinction in immune response between natural infection and vaccination for SARS-CoV-2 can provide important insights for vaccine strategies and for the biology of coronaviruses.

The aim of the study was to explore the correlation between the avidity index and neutralization titer, examine whether the avidity index of anti-spike IgG can contribute to a better and more precise estimation of the neutralizing capacity on SARS-CoV-2 virus, and also to investigate the immune response after primary infection with SARS-CoV-2, compared to two doses of vaccine.

## 2. Methods

### 2.1. Samples

Serum samples were sourced from patients in two cohorts in an ongoing study in Halland, Sweden, “COVID-19—symptoms and immunity.” This study is a prospective study with one cohort consisting of patients with nonsevere COVID-19 infection during June–August 2020 (*n* = 75) and one cohort consisting of COVID-19 naïve patients (*n* = 39) who were vaccinated against COVID-19 during April–August 2021. To be included in the COVID-19 cohort, a positive PCR-test for SARS-CoV-2 was required. The patients in the vaccine cohort were included during March 2021, and to be included, a negative test for SARS-CoV-2 nucleocapsid antibodies and no ongoing symptoms of COVID-19 were required. Serum samples were taken 1, 3, and 6 months after the infection, or after the second dose of vaccine, depending on the cohort. Due to logistical reasons, only a total of 80 serum samples could be analyzed in the neutralization assay and the 6-month samples from the vaccination cohort were not available at that time. For this reason, samples from both cohorts at the different time points were sought and patients with samples from all time points were prioritized. The study was approved by the Swedish Ethical Review Authority, approval number Drn: 2020–02691 and 2021–00355, and all patients gave informed consent to participate.

### 2.2. SARS-CoV-2 Antibody Detection and Avidity Index

For analyzing anti-spike IgG, Anti-SARS-CoV-2 QuantiVac ELISA (Euroimmun) was performed according to the manufacturer's instruction. Results of ≥ 11 RU/mL were considered as positive, ≥ 8–< 11 RU/mL as borderline, and < 8 RU/mL were considered negative according to the manufacturer's cutoffs. Regarding the avidity analysis, the same assay was used but an extra step with a chaotropic agent was added, as previously described [17]. Briefly, the samples were added in duplicates to the wells and, after the first incubation, an extra step was included where 300 μL of buffer solution with 4M urea was added to one well and 300 μL of pure buffer solution was added to the other well. After 10 min, the rest of the procedure was carried out according to the manufacturer's instruction except for the modification with two washing cycles instead of one. The avidity index was defined as OD_urea_/OD_reference_ and multiplied by 100 to be expressed as percentage. If the IgG level was above the linear range, the sample was diluted to fit in the linear range of the method and reanalyzed. Two serum samples were used as positive controls throughout all the analyses and the coefficient of variability (CV) for the avidity index of the two controls were 12% and 8%. To combine the antibody level and avidity index, a product was created by multiplying the antibody level (RU/mL) with the avidity index (%).

### 2.3. Neutralizing Capacity

Serum neutralization titer was analyzed in a cytopathic effect (CPE)–based microneutralization assay, described by Varnaite et al. [18]. After 1 h of incubation, the serum–virus mixture was added in duplicates to Vero E6 cells and incubated for 5 days, and then wells were inspected with optical microscopy for signs of CPE and classified as either neutralizing (less than 50% CPE) or nonneutralizing. As a result, the neutralization titer of the reciprocals of the highest neutralizing dilutions from the two duplicates for each sample was used.

### 2.4. Statistics

Statistical analysis utilized SPSS Version 29, and since the data were not normally distributed, nonparametric tests were used for further calculations. Spearman correlation was used for the correlation analysis. When calculating differences in antibody levels, avidity index, and neutralization titer between different cohorts, Mann–Whitney U-test was used, and for differences over time within the cohorts, Wilcoxon signed-rank test was used. A *p* value of < 0.05 was considered significant.

## 3. Results

In the neutralization assay, 80 serum samples were analyzed but since samples from one patient could not yield results in the neutralization assay, 79 serum samples from 34 patients were used for further calculations. For anti-spike levels and avidity index, 12 additional samples from the same included patients were analyzed. All serum samples were taken 1, 3, and 6 months (±7 days) after the PCR-confirmed COVID-19 (infection cohort) or after the second dose of vaccine (vaccine cohort).

Mean age of patients in the total population was 46 years old (range 21–69 years). 68% (*n* = 23) were women and 38% (*n* = 13) were in the infection cohort. All but one patient in the vaccine cohort were vaccinated with mRNA vaccine BNT162b2 (Pfizer/BioNTech). There were no differences in mean age (47 years vs. 46 years) between the two cohorts, but the proportion of women was higher in the vaccine cohort than in the infection cohort (76% (*n* = 16) vs. 54% (*n* = 7)). Based on surveillance data, B.1 and B1.1 were the dominating virus variants in the county of Halland during the inclusion period for the infection cohort.

**Figure 1:**
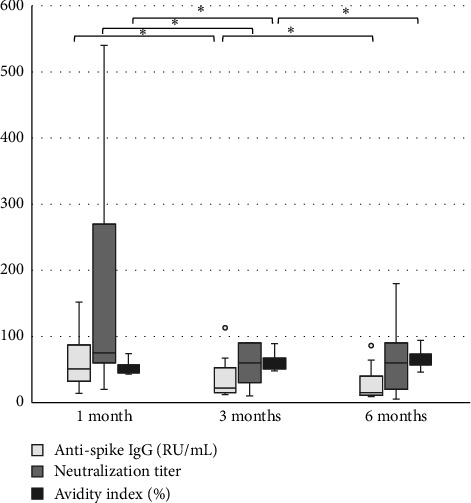
Levels of anti-spike IgG (RU/mL), neutralization titer, and avidity index (%) after PCR-confirmed COVID-19 (infection cohort). Wilcoxon signed-rank test is used for calculating differences over time, ^∗^*p* < 0.05.

**Figure 2:**
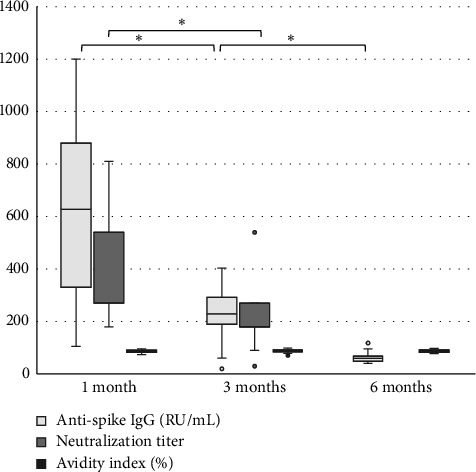
Levels of anti-spike IgG (RU/mL), neutralization titer, and avidity (%) after two doses of vaccine against SARS-CoV-2 (vaccine cohort). Wilcoxon signed-rank test is used for calculating differences over time, ^∗^*p* < 0.05.

**Figure 3:**
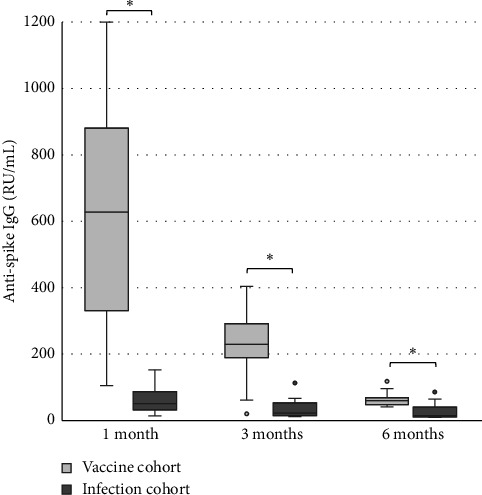
Levels of anti-spike IgG (RU/mL) after two doses of vaccine against SARS-CoV-2 (vaccine cohort) and after PCR-confirmed COVID-19 (infection cohort). Mann–Whitney U-test is used for calculating differences between the cohorts, ^∗^*p* < 0.05.

**Figure 4:**
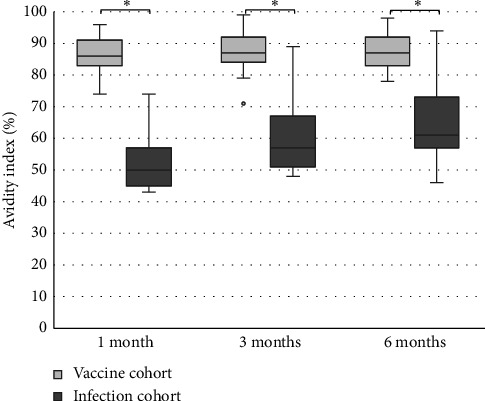
Avidity index (%) after two doses of vaccine against SARS-CoV-2 (vaccine cohort) and after PCR-confirmed COVID-19 (infection cohort). Mann–Whitney U-test is used for calculating differences between the cohorts, ^∗^*p* < 0.05.

**Figure 5:**
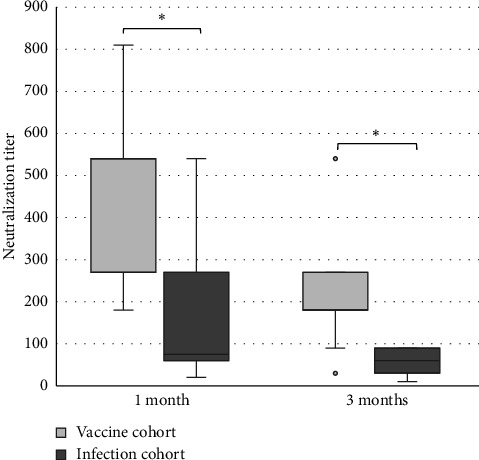
Neutralization titer after two doses of vaccine against SARS-CoV-2 (vaccine cohort) and after PCR-confirmed COVID-19 (infection cohort). Mann–Whitney U-test is used for calculating differences between the cohorts, ^∗^*p* < 0.05.

The levels of anti-spike IgG (RU/mL), avidity index (%), and neutralizing titer at 1, 3, and 6 months after infection or after the second dose of vaccine (infection cohort and vaccine cohort) are presented in Figures 1 and 2 and Table 1. There was a significant difference (*p* < 0.05) in the levels between the two cohorts at all time points with higher levels of anti-spike, avidity index, and neutralizing capacity in the vaccine cohort, see Figures 3, 4, and 5. Within the two cohorts, the anti-spike IgG levels and the neutralization titer decreased significantly (*p* < 0.05) over time, see Figures 1 and 2. Regarding the avidity index, this did not change significantly in the vaccine cohort but increased significantly, with an increase in the median value of 11% (*p* < 0.05) over time, in the infection cohort.

Using Spearman correlation, a significant correlation between level of anti-spike IgG (RU/mL) and neutralization titer was found, Spearman *r*_*s*_ = 0.88, *p* < 0.001. Between the avidity index and neutralization titer, the correlation was lower, but still significant, Spearman *r*_*s*_ = 0.62, *p* < 0.001. When combining the antibody levels with the avidity index as a product, the correlation coefficient was Spearman *r*_*s*_ = 0.89, *p* < 0.001. The results for different subgroups (infection/vaccinated, anti-spike IgG > 80 RU/mL, anti-spike IgG < 40 RU/mL, and high-avidity antibodies) are presented in Table 2.

## 4. Discussion

The aim of our study was to explore if avidity can contribute to a better estimation of the neutralizing capacity. If so, avidity could be used as a more practical method to assess immunity in individual patients and also to predict risk for severe COVID-19 and efficacy/protection from vaccination.

The results show a strong and significant correlation between the anti-spike IgG levels and the neutralization titer, which is in line with other studies [4, 19]. However, the addition of the avidity index did not yield a better correlation. The avidity alone had a significant correlation with the neutralization titer, but it was weaker than the correlation between neutralization titer and the antibody levels alone. There was no difference in correlations between the two cohorts, vaccination and natural infection.

Dapporto et al. suggest that only antibodies with high avidity are involved in neutralization, but we could not find any stronger correlation with neutralizing capacity in the subgroup of sera with avidity index > 75% [14]. Reasonably, even though there is no scientific evidence, the avidity may have a greater impact for the neutralizing capacity when the levels of antibodies are low. To test this hypothesis, the correlations between avidity and neutralization titer in subgroups of different anti-spike IgG levels were calculated. The results failed to show a difference, with a weak and nonsignificant correlation in the subgroups with low antibody levels (< 80 RU/mL and < 40 RU/mL). According to these results, the avidity index is not a good surrogate marker for neutralizing capacity, independent of antibody level. High levels of anti-spike IgG seem to be a more important factor for neutralizing SARS-CoV-2. Still, avidity can be an important part of developing long-lasting and protective immunity since high-avidity antibodies have been shown to achieve a broader recognition of SARS-CoV-2 epitopes and an increased capacity to neutralize SARS-CoV-2 variants of concern [8, 20].

Interestingly, the results confirm that the anti-spike IgG levels alone can be used as an estimation of the neutralization capacity. In our experience, this is an assumption that is already used in everyday clinical work and our results justify this practice. Despite this, a challenge is to determine if there is a reliable cutoff for a protective level of antibodies [21, 22]. This issue is out of the scope of the present study since it requires clinical data of reinfection as well as patients with both mild and severe COVID-19. So far, other studies have shown that seronegativity is a major risk for severe COVID-19 but no cutoff has been proposed [23]. Notably, the results in this study also showed that in patients with low levels of anti-spike IgG (< 40 RU/mL), the correlation with neutralizing titer, even though it was significant, was much weaker. This implies that it is harder and more uncertain to estimate the neutralizing capacity using the levels of anti-spike IgG when they are low. This finding could be one of the reasons for the difficulties of finding a clinically useful cutoff. Also, since other parts of the immune system, such as T-cells response and cytokines, are important to clear a virus infection, it might be difficult to determine a cutoff for protective immunity based solely on antibody levels.

Our findings also showed a significantly greater immune response after the second dose of vaccine compared to natural infection. The result in our study showed that the anti-spike IgG levels, the avidity index as well as the neutralization titer, were higher in the vaccine cohort. Unfortunately, there was no possibility to analyze the neutralization titer in the vaccine cohort at 6 months after the vaccination in this study, due to logistical issues. Still, the results in the vaccine cohort clearly indicate that high-avidity antibodies had developed already after 1 month and the avidity index did not decrease after 6 months. The differences compared to the infection cohort were still significant after 6 months. This result also shows that natural infection failed to induce high avidity antibodies since the median avidity index in the infection cohort never exceeded 61%, in line with other studies [14, 24–26]. One explanation might be that a natural nonsevere infection does not give rise to high levels of antigen in the blood, since the virus is primarily located in the airways, in contrast with vaccination or severe COVID-19 [15]. The pattern with low avidity maturation after infection may also be a feature of coronavirus since incomplete avidity maturation can be seen in seasonal coronavirus infections too [27].

For the antibody levels, there is a significant decrease over time, seen already 3 months after both infection and vaccination but more pronounced in the vaccine cohort. Interestingly, at 6 months, the difference between the cohorts was reduced so much that the difference is reasonably not clinically relevant. The neutralizing titer shows a similar pattern, with diminishing titers already at 3 months and less difference between the cohorts over time. Others have similarly reported declining levels of antibodies and neutralization titer after both infection and vaccination [25, 26, 28–30]. These findings support the need of a vaccination booster and also support the recommendation of vaccination despite previous SARS-CoV-2 infection.

The study has several limitations. As mentioned before, we include only samples from patients with mild/nonsevere COVID-19 in this study and there are no immunosuppressed patients in any of the cohorts. Other studies have shown different antibody kinetics in patients with severe or hospitalized COVID-19, having higher antibody levels and higher avidity [15, 16]. This might have affected the results, which therefore cannot be generalized to patients with severe COVID-19 or immunocompromised patients. Nonetheless, these results should be representative of the vast majority of SARS-CoV-2 infection. Also, in the study population, the majority are female and the proportion of females in the vaccine cohort is considerably higher than that in the infection cohort. Previous studies have shown differences in the immune response between men and women, and it has also been shown that men are at higher risk of severe COVID-19 [25, 29, 31]. The number of participants was unfortunately too small to separate the results between men and women, since the different time points and different cohorts of vaccine or infection had to be taken into the calculations. However, an earlier study showed that the differences between men and women, when it comes to antibody levels and avidity, were small and not clinically significant [17].

In conclusion, in nonsevere COVID-19, the levels of anti-spike IgG, especially high levels, can be used as an estimation of the neutralizing titer. The avidity index did not contribute to a stronger correlation or better estimation. There is a stronger initial immune response with high avidity maturation after vaccination compared to natural infection. However, over time, antibody levels and neutralization titer, as well as the difference between the vaccination and the infection cohorts, declined, emphasizing the importance of vaccine boosters.

## Figures and Tables

**Table 1:** Median value (range) of anti-spike IgG, avidity index, and neutralizing titer at different time points after vaccination or after COVID-19.

	1 month	3 months	6 months
*Anti-spike IgG (RU/mL)*			
Infection cohort	51 (14–152) *n* = 13	22 (12–113) *n* = 13	15 (9–86) *n* = 13
Vaccination cohort	628 (105–1200) *n* = 21	229 (20–404) *n* = 21	60 (16–118) *n* = 11

*Avidity index (%)*			
Infection cohort	50 (43–74) *n* = 13	57 (48–89) *n* = 13	61 (46–94) *n* = 13
Vaccination cohort	86 (74–96) *n* = 21	87 (71–99) *n* = 21	87 (78–98) *n* = 11

*Neutralizing capacity (titer)*			
Infection cohort	75 (20–540) *n* = 12	60 (10–90) *n* = 13	60 (5–180) *n* = 13
Vaccination cohort	540 (180–810) *n* = 20	180 (30–540) *n* = 21	—

*Note: n* = numbers of patients.

**Table 2:** Correlation (Spearman's *r*_*s*_) with neutralizing titer in different subgroups.

	Total cohort (*n* = 79)	Infection cohort (*n* = 38)	Vaccine cohort (*n* = 41)	Cohort anti-spike > 80 RU/mL (*n* = 44)	Cohort anti-spike < 80 RU/mL (*n* = 35)	Cohort anti-spike < 40 RU/mL (*n* = 24)	Cohort avidity index > 75% (*n* = 43)
Anti-spike IgG	0.88^∗^	0.79^∗^	0.78^∗^	0.66^∗^	0.68^∗^	0.49^∗^	0.82^∗^
Avidity index	0.62^∗^	0.22	0.32^∗^	0.31^∗^	0.29	0.08	0.33^∗^
Combined product (anti-spike multiplied with avidity index)	0.89^∗^	0.80^∗^	0.81^∗^	0.69^∗^	0.67^∗^	0.49^∗^	0.84^∗^

*Note: n* = number of blood samples.

^∗^ *p* < 0.05.

## Data Availability

Data are available on request by emailing to emma.lofstrom@med.lu.se.
